# Virome Analysis of Signal Crayfish (*Pacifastacus leniusculus*) along Its Invasion Range Reveals Diverse and Divergent RNA Viruses

**DOI:** 10.3390/v13112259

**Published:** 2021-11-11

**Authors:** Katarina Bačnik, Denis Kutnjak, Silvija Černi, Ana Bielen, Sandra Hudina

**Affiliations:** 1 Department of Biotechnology and Systems Biology, National Institute of Biology, 1000 Ljubljana, Slovenia; katarina.bacnik@nib.si; 2 Jozef Stefan International Postgraduate School, 1000 Ljubljana, Slovenia; 3 Department of Biology, Faculty of Science, University of Zagreb, 10000 Zagreb, Croatia; silvija.cerni@biol.pmf.hr; 4 Department of Biochemical Engineering, Faculty of Food Technology and Biotechnology, University of Zagreb, 10000 Zagreb, Croatia; abielen@pbf.hr

**Keywords:** signal crayfish virome, RNA viruses, invasive alien species, invasion range, high-throughput sequencing

## Abstract

Crayfish are a keystone species of freshwater ecosystems and a successful invasive species. However, their pathogens, including viruses, remain understudied. The aim of this study was to analyze the virome of the invasive signal crayfish (*Pacifastacus leniusculus*) and to elucidate the potential differences in viral composition and abundance along its invasion range in the Korana River, Croatia. By the high-throughput sequencing of ribosomal RNA, depleted total RNA isolated from the crayfish hepatopancreas, and subsequent sequence data analysis, we identified novel and divergent RNA viruses, including signal crayfish-associated reo-like, hepe-like, toti-like, and picorna-like viruses, phylogenetically related to viruses previously associated with crustacean hosts. The patterns of reads abundance and calculated nucleotide diversities of the detected viral sequences varied along the invasion range. This could indicate the possible influence of different factors and processes on signal crayfish virome composition: e.g., the differences in signal crayfish population density, the non-random dispersal of host individuals from the core to the invasion fronts, and the transfer of viruses from the native co-occurring and phylogenetically related crayfish species. The study reveals a high, previously undiscovered diversity of divergent RNA viruses associated with signal crayfish, and sets foundations for understanding the potential risk of virus transmissions as a result of this invader’s dispersal.

## 1. Introduction

Until recently, knowledge of invertebrate viruses was limited mostly to viral pathogens causing high mortalities and arboviruses (arthropod-borne viruses), which are vectored by arthropods and cause disease in humans and other vertebrate species [1]. This view has changed, with metagenomics revealing remarkable levels of RNA virus diversity in invertebrates [2,3,4,5], and, in this perspective, the detection of invertebrate disease-causing viruses became the exception rather than the rule [1]. In addition to transforming our understanding of virus diversity, virus metagenomics provides insights into different aspects of virus evolution [6], including the role of invertebrates and their ecological interactions with other organisms in the evolution of RNA viruses [5].

For understanding the diversity of invertebrate viruses, arthropods, including crustaceans, may be of particular importance, as they account for over 80% of the total animal diversity [7], are globally abundant, and often live in extremely large and dense populations [6].

Crayfish, belonging to a diverse order of decapod crustaceans, are a keystone species of freshwater ecosystems, and their populations can reach high densities. They are an important component of freshwater food webs and ecosystem engineers due to their larger size and longer life span compared to other benthic macroinvertebrates, as well as due to their bioturbation and burrowing activity [8]. Crayfish are also among the most successful invasive alien species (IAS) in freshwater ecosystems [9]. Throughout history, crayfish have been frequently translocated for ornamental or aquaculture purposes [10] and, after being intentionally or unintentionally released into the wild, have often rapidly expanded their range and exerted a high number of documented negative impacts [10,11]. On the other hand, native crayfish populations in the wild are increasingly endangered on the global scale [9,10,12,13], and invasive crayfish and their pathogens have been identified as one of the most prominent factors contributing to the decline of native species’ populations [14,15]. Some of the pathogens introduced by invasive crayfish are significantly more virulent towards the new (i.e., native) hosts than toward IAS hosts [16,17]. Thus, invasive crayfish species have a high potential for introducing and spreading the emerging diseases that may incur significant economic and ecological losses [18,19]. However, the existing literature on crayfish diseases is biased mostly towards the crayfish plague pathogen, the oomycete *Aphanomyces astaci*, while other disease agents, including fungi, bacteria, and viruses, are significantly understudied [20].

Most of the knowledge on crustacean pathogenic viruses comes from the study of viral diseases in aquaculture [20]. For instance, white spot syndrome virus causes the white spot disease that has mostly been described due to its symptoms in aquaculturally relevant species such as *Cherax quadricarinatus* [21], although it displays a low degree of host specificity, and is therefore also an important pathogen in crayfish populations in the wild [22]. Despite their large potential impact on aquaculture and populations in the wild, viruses are the least studied group of crayfish pathogens [20], and relatively few have been formally characterized and classified [23]. However, with the increasing availability of high-throughput sequencing (HTS) technologies and metagenomics approaches, which can reveal all of the viruses (i.e., virome) associated with an individual or an environmental sample, comprehensive descriptions and a revolution in our understanding of the viral diversity of crustacean species are occurring [23]. For instance, this approach was recently employed to discover bunya-like brown spot virus, a new negative, single stranded RNA virus belonging to the *Phenuiviridae* family that was associated with a massive disease outbreak in the population of the endangered white-clawed crayfish *Austropotamobius pallipes* [24].

The aim of our study was to analyze the virome of the signal crayfish, *Pacifastacus leniusculus*, (Dana, 1852), one of the most successful freshwater invaders in Europe. The signal crayfish is present in over 29 EU countries [25], reaches very high dispersal rates within European watercourses [26], and is listed as IAS of Union concern (EU Regulation No. 1143/2014 on invasive alien species). We have investigated the signal crayfish population in a recently invaded Korana river, Croatia, where signal crayfish range expansion is well monitored [27,28,29], and occurs in both upstream and downstream directions [27]. Recently established populations at invasion fronts have 6–11 times lower crayfish abundance in comparison to longer established populations in invasion cores, and also contain co-occurring native congener (narrow-clawed crayfish, *Pontastacus leptodactylus*, Eschscholtz, 1823) [27]. Host population density, with increased contact rates among individuals in highly dense populations, is an important factor affecting rates of virus transmission [30,31].

In this study, we sampled the signal crayfish individuals at four locations along its invasion range, including upstream and downstream invasion cores and invasion fronts. We sequenced and analyzed signal crayfish samples to: (1) identify putative viral sequences in the signal crayfish hepatopancreas, as this is the tissue most often associated with viral infections in freshwater crayfish [20]; (2) describe the phylogenetic relationships of the most abundant novel signal crayfish-associated viruses; (3) discuss the potential differences in virome composition and the abundance of viral sequences along the invasion range. Our results reveal a high, previously undiscovered diversity of viruses in signal crayfish, with putative signal crayfish-associated reo-like, hepe-like, toti-like, and picorna-like viruses, representing divergent sequences most similar to the viruses previously associated with crayfish hosts.

## 2. Materials and Methods

### 2.1. Study Area and Sample Collection

**Figure 1:**
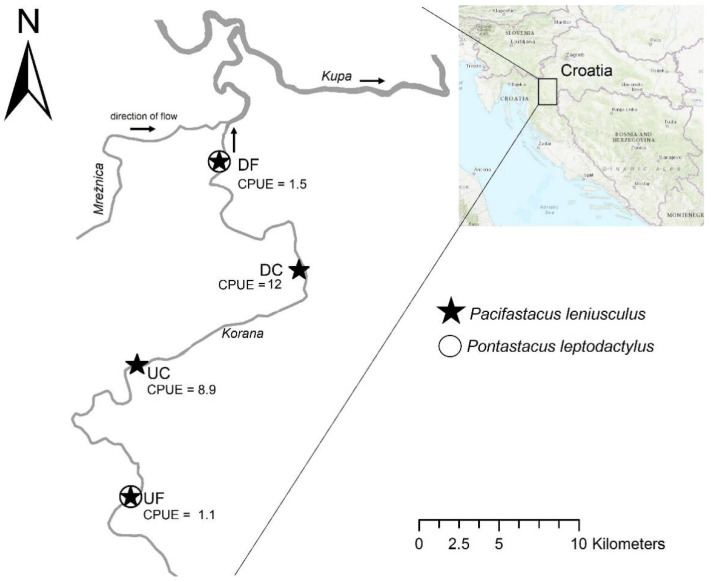
Position of sampling locations and differences in crayfish (*Pacifastacus leniusculus* and *Pontastacus leptodactylus*) presence and abundance (CPUE; catch per unit effort) along the signal crayfish invasion range in the Korana River in 2018. Sampling was performed at both upstream (UF) and downstream (DF) invasion fronts and upstream (UC) and downstream (DC) invasion cores.

Research was carried out in the continental part of Croatia, in the Korana River. Here, the signal crayfish was illegally introduced in the lower section of the river, and is spreading both upstream and downstream [28], with its invasion range currently stretching along 33 km [27]. Significant differences in crayfish abundance occur along the signal crayfish invasion range, i.e., between the longer established populations in the center of its distribution (invasion core) compared to the recently established populations at distribution edges (invasion fronts). Invasion front locations have up to 11 times lower crayfish abundance compared to the invasion core [27]. Also, at invasion fronts, interspecific populations of the signal crayfish and the native narrow-clawed crayfish (*Pantastacus leptodactylus*, Eschscholtz, 1823) are present, with 2.5–12.9 higher abundances of the native *P. leptodactylus* compared to the signal crayfish [27]. Populations in the invasion cores comprise only signal crayfish, which have gradually displaced the native *P. leptodactylus* [27,29], as depicted in Figure 1.

Crayfish were collected in the period of increased crayfish activity (early autumn 2018) using baited LiNi traps [32]. A total of 110 signal crayfish individuals were captured from four invasion range endpoints (Figure 1), which were previously [27] identified as: the upstream invasion front (UF), upstream invasion core (UC), downstream invasion core (DC), and downstream invasion front (DF). For each location, we calculated the catch per unit effort (CPUE) that equals the number of crayfish captured per each trap per trapping night, and is a frequently used measure of relative crayfish abundance (Figure 1). The captured indigenous crayfish species were returned to the same location where they were caught, while signal crayfish individuals from all four sampling locations were taken on ice to the laboratory where hepatopancreas samples were randomly collected from 25 individuals from each location. For each individual, the complete organ was removed from the body, placed in a sterile Petri dish, and carefully chopped into smaller pieces using a sterile scalpel. The cut pieces of the organ were stored at −80 °C in an RNA stabilizing agent (RNA later; Sigma Aldrich, MO, USA) until RNA extraction.

### 2.2. RNA Extraction and Sequencing

The total RNA was extracted from hepatopancreas tissue using the RNeasy Lipid Tissue Mini Kit (Qiagen CA, USA). Before RNA extraction, equal amounts of tissue (30 mg) of each of the 25 individuals collected at the same location were pooled together, resulting in four composite pools corresponding to four sampling locations. After homogenizing tissue in liquid nitrogen, RNA extraction, followed by on-column DNase digestion step (RNase-Free DNase Set, Qiagen), was performed as recommended by the manufacturer. The eluted RNA from all four samples was sent for ribosomal RNA depletion (Illumina Ribo-Zero Plus rRNA Depletion Kit), sequencing library preparation (NEBNext Ultra RNA Library Prep Kit), and shotgun sequencing (Illumina HiSeqX, 2 × 150 bp) to CD Genomics, USA.

### 2.3. Bioinformatic Analysis for Detection of Viral Sequences

Sequencing datasets were quality checked, filtered, and analyzed as described below. The overview of the bioinformatic analyses performed is schematized in Supplementary material, Figure S1. The remains of the sequencing adaptors were trimmed, and resulting reads were filtered by using a quality filter (Limit = 0.01; no ambiguous nucleotide allowed) and by size (reads shorter than 25 bp were discarded) in CLC Genomics Workbench 20 (Qiagen Bioinformatics, CA, USA). Trimmed, size, and quality filtered reads were exported from the CLC Genomics Workbench and compared for similarity with a complete NCBI nr database (2020) using Diamond blastx [33] with default parameters, followed by the taxonomic classification of the reads and visualization of the results using Megan (Metagenome Analyzer, version 6.20.19, Tübingen, Germany) [34].

To identify longer virus-like sequences, reads were used for de novo assembly using SPAdes (version 3.14.0, St. Petersburg, Russia) [35], and the de novo constructed contigs were queried for similarity on protein level using Diamond blastx [33], followed by the taxonomic classification of the reads and visualization of the results using Megan [34]. The contig sequences that were classified as viral in all four analyzed samples were imported into the CLC Genomics Workbench, where additional steps of de novo assembly of viral contigs from all four samples was performed, resulting in a further assembly of overlapping sequences from different samples. The contigs that were not classified as viral by Diamond-Megan analysis were further analyzed using conserved domain search against Pfam (v32) database, and additional virus-like sequences detected using this analysis were added to the list of virus-like sequences from Diamond-Megan analysis.

The contigs that were identified as virus-like sequences according to the described analysis (Figure S1) were further investigated using blastn (NCBI-nt, April 2021) analysis to identify any possible misclassified sequences. After the additional manual inspection of all the similarity searches, we classified virus-like sequences into different categories (Table S2). Non-viral hits, such as host sequences misclassified as viral sequences (similar to viruses from *Nudiviridae* family) and possible endogenized virus-like sequences (e.g., members of *Retroviridae* and *Adintoviridae* families), as well as viral sequences belonging to bacteriophages (e.g., members of *Caudovirales* order, *Leviviridae* family), plant infecting viruses (e.g., members of *Solemoviridae* and *Bromoviridae* families), and sequences identified as laboratory contamination (e.g., *Cryphonectria hypovirus*) were excluded from further analysis. The final selection of virus-like contigs included only sequences of potential invertebrate viruses longer than 300 nts. The differences between the abundance of reads corresponding to selected virus-like contigs detected in individual samples from different locations were identified by mapping the reads to virus-like contig sequences in CLC Genomics Workbench 20 (length fraction = 0.90, similarity fraction = 0.90), and the percentage of mapped reads was calculated (Table S1). In order to normalize samples according to the number of reads before mapping them to contig sequences, random subsampling was done and all samples were normalized to 97,563,944 reads, which was the minimum number of reads obtained among the samples. Decimal logarithm transformed average read coverage values were visualized as a heatmap, and used for clustering the locations and viruses using the heatmap.2 function in a gplots package in R 4.0.5 [36] 

All sequencing data that support the findings of this study are linked under the accession number PRJNA754774 in the NCBI BioProject database. The sequences of selected viral contigs identified in this study are available in Table S2 and have been deposited in the GenBank under the accession numbers OK317706-OK317734.

### 2.4. PCR Detection of Selected Viral Contigs in Hepatopancreas Samples

Based on the presence of RNA-dependent RNA polymerase (RdRp) domain, contig length, and average read coverage, we selected four viral contigs for further analysis; virus-like contig 4 (signal crayfish-associated reo-like virus 1), virus-like contig 139 (signal crayfish-associated hepe-like virus 1), virus-like contig 1 (signal crayfish-associated picorna-like virus 1), and virus-like contig 141 (signal crayfish-associated toti-like virus 1). To confirm their presence in the samples, primer pairs (Table S3) amplifying the fragments of newly detected sequences were constructed using Primer-BLAST [37]. cDNA was synthesized from 2 µL of total RNA in a reaction volume of 20 µL by random priming using a High-Capacity cDNA Reverse Transcription Kit (Applied Biosystems, CA, USA) as described by the manufacturer, using the following reaction conditions: 25 °C for 10 min, 37 °C for 120 min, and 85 °C for 5 min. Target fragments were amplified in a total volume of 25 µL using 1 µL of cDNA template, 2 mM MgCl_2_, 0.2 mM dNTP mixture, 0.5 µM each primer, and 0.0625 U of G2 polymerase (GoTaq Flexi DNA Polymerase, Promega, WI, USA) and following cycling conditions: 95 °C for 2 min, 40 cycles of 95 °C for 30 s, 58 °C for 30 s, and 72 °C for 30 s; followed by 5 min of final extension at 72 °C. Amplicons were detected after electrophoresis in 1% agarose gel stained with SYBR Safe DNA Gel Stain (Invitrogen, CA, USA).

### 2.5. Phylogenetic Analyses

For selected virus-like contigs 4, 139, 1, and 141, the most similar homologous sequences with corresponding RdRp sequences were retrieved from the NCBI using blastp search against NCBI nr database (May 2021), and used in the phylogenetic analysis. The RdRp protein sequences of recognized viral species belonging to reo-like, hepe-like, picorna-like and toti-like virus groups selected according to previous studies [3] were additionally included in the phylogenetic analysis from the GenBank database. RdRp amino acid sequences were aligned using the L-INS-I algorithm of the MAFFT program [38], and alignments were trimmed manually and with TrimAI (automated 1 mode) [39]. Alignments were visualized in MEGAX [40]. Sequences included into the alignments with corresponding GenBank accession numbers are given in Alignments S1–S4. Maximum likelihood trees were inferred using PhyML (v. 3.00, Montpellier, France) [41] available in the Phylemon 2.0 web server [42] employing the Le Gascuel (LG + I + G) amino acid replacement model, selected by IQTree ModelFinder [43], using 1000 bootstrap replicates.

### 2.6. Analysis of Nucleotide Diversities

To compare the possible differences in the diversities of populations of selected viruses detected at all sampling locations, we determined the nucleotide diversities of populations of three signal crayfish-associated viruses, which were present at all sampling locations with an average read coverage higher than 40. For these analyses, read data was additionally filtered. First, reads with low nucleotide quality (<20) were discarded using a FASTQ Quality Filter (FASTX-toolkit). Reads from individual samples were then mapped to selected viral contigs (length fraction = 0.90, similarity fraction = 0.90). Before mapping, the read datasets were subsampled to amount for the same read coverage of individual contig sequence in different samples (higher read coverage was normalized by using a lower number of reads per sample). Single-nucleotide polymorphism (SNP) calling was performed using a Low Frequency Variant Detection tool in the CLC Genomics Workbench 20 (required significance = 1%, minimum coverage = 10, minimum count = 2, minimum frequency = 1%). SNP tables were used as an input for SNPGenie software [44], which was used to calculate diversity indices for each viral contig—location combination. Calculated nucleotide diversity (π) values, which represent the mean number of pairwise differences per nucleotide in a population of mapped reads, were plotted and compared according to the location.

## 3. Results and Discussion

### 3.1. Novel Viral Sequences Identified in Hepatopancreas of Signal Crayfish

#### 3.1.1. Overview of the Newly Identified Viral Sequences in Hepatopancreas of Signal Crayfish

To identify viral sequences, we performed sequencing of ribosomal RNA depleted total RNA resulting in high numbers (100–164 M) of reads per sample (Table 1). First, in a reads-based protein similarity search, a small fraction of reads (at most, 0.018%) was classified as viral sequences (Table 1). Read sequences similar to different RNA viruses (classified as Riboviria) were the most abundant viral sequences in all four samples. Our analysis of de novo assembled contigs, presented in Table S2 together with blast resulting similarities and average read coverage values, supported the observed presence of sequences similar to different unclassified RNA viruses. Results revealed the presence of diverse virus-like sequences, with selected virus-like contigs (longer than 300 nts) representing sequences associated with invertebrate hosts shown in Table 2.

Sequences belonging to potential invertebrate viruses exhibited relatively low levels of similarity to known viral sequences, with sequence identities ranging from 25% to 84% according to blastx results. Many virus-like contigs representing novel, highly divergent RNA viral sequences were most similar to reo-like, hepe-like, toti-like, picorna-like, tombus-like, chu-like, and partiti-like viruses. A small fraction of viral reads and the dominance of previously unknown RNA viruses in aquatic arthropod hosts samples is in line with previous studies [3,5,45]. Many of the virus-like sequences identified in this study had the highest identities with unclassified RNA viruses discovered as part of a large-scale metagenomics study of invertebrate viromes [3], where similarities with viruses described in different crustacean hosts were also observed.

**Figure 2:**
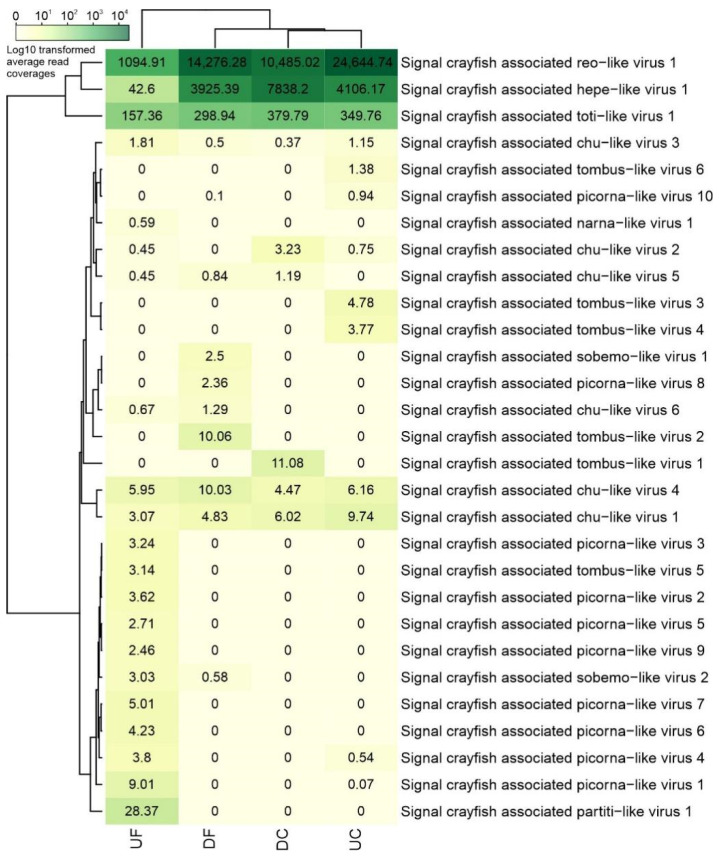
Heatmap showing average read coverage resulting from mapping of normalized read datasets from individual locations to selected putative invertebrate virus-like contigs together with the dendrograms clustering the sampling locations (top) and viral contigs (left) according to average read coverage values. Heatmap and dendrograms were constructed using decimal logarithm transformed values, however, non-transformed average read coverage numbers are plotted on the heatmap.

Some viral contig sequences (virus-like contigs 139, 141) discovered in this study likely correspond to full-length genomes, given that the length and genomic organization is similar to the annotated closest hits from the blastx analysis. High average read coverage values were obtained for some virus-like contigs (Figure 2), which may further support the assumption that they represent crayfish infecting viruses. However, confidently assigning host associations based on metagenomics studies and bioinformatic analyses remains a challenge [1], and therefore, some virus-like contigs identified here may be associated with other organisms that are present within the studied host [46].

#### 3.1.2. Phylogenetic Relationships and Genome Organization of Selected Newly Identified Viral Sequences

**Figure 3:**
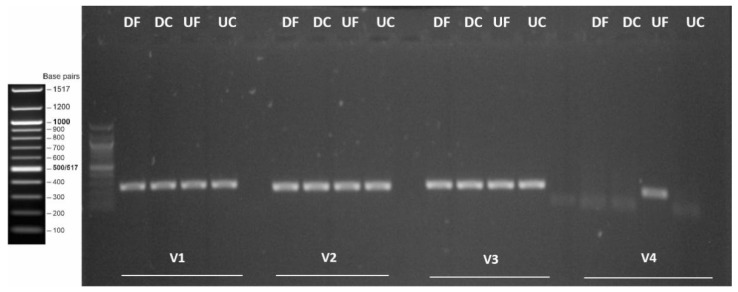
Gel electrophoresis of PCR amplicons originating from newly identified signal crayfish-associated viruses. Four primer pairs were used for amplifying app. 200 nts long regions of signal crayfish-associated reo-like virus 1 (V1), signal crayfish-associated hepe-like virus 1 (V2), signal crayfish-associated toti-like virus 1 (V3), and signal crayfish-associated picorna-like virus 1 (V4) genomes. Samples from different locations (UF—upstream front, UC—upstream core, DC—downstream core, DF—downstream front) were analyzed. Unmarked wells represent no template controls of each amplification reaction.

Based on the presence of the RdRp domain and contig length, we selected four distinct viral contigs for further analyses. They represent putative novel virus species belonging to reo-like (virus-like contig 4), hepe-like (virus-like contig 139), toti-like (virus-like contig 141), and picorna-like (virus-like contig 1) virus clades. These viral sequences showed similarities to viruses previously described in crustacean hosts, suggesting that they were likely infecting signal crayfish rather than being associated with the environment or co-infecting microorganisms. The presence of signal crayfish-associated reo-like virus 1, hepe-like virus 1, and toti-like virus 1 sequences was confirmed in all four analyzed samples by obtaining the products of the expected size after RT-PCR (Figure 3), while the presence of signal crayfish-associated picorna-like virus 1 was confirmed in the UF sample only, in line with the sequencing results (Figure 2).

**Figure 4:**
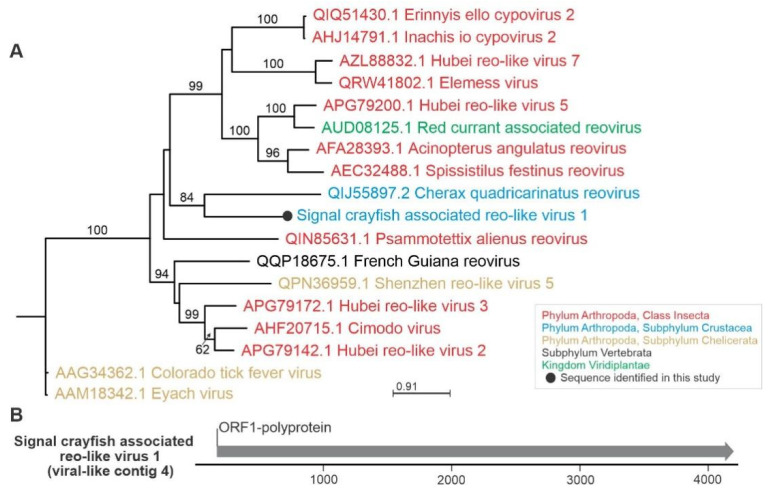
Phylogenetic relationships and genome organization of signal crayfish-associated reo-like virus 1. (**A**) Phylogenetic tree built with maximum likelihood approach, based on the alignment of the conserved segment of RNA-dependent RNA polymerase domain of signal crayfish-associated reo-like virus 1 (virus-like contig 4) and representative selected sequences of phylogenetically related viruses. The numbers on the branches represent bootstrap support values (>50% shown), the branch length represents the average number of amino acid substitutions per site (**B**) The predicted partial genome organization of the novel signal crayfish-associated reo-like virus 1, with positions and length of the open reading frames (ORF) indicated with corresponding arrow length.

In our study, signal crayfish-associated reo-like virus 1 (virus-like contig 4, 4234 nts) had the highest average read coverage in all samples (Figure 2). A variety of reoviruses have been described previously in crustacean hosts [23]. Using blastx, we observed a distant similarity to the RdRp partial coding sequence of Cherax quadricarinatus reovirus, which was recently associated with hepatopancreatic samples in redclaw crayfish (*Cherax quadricarinatus*) from Australia [47]. This relationship was also confirmed by phylogenetic analysis (Figure 4). Cherax quadricarinatus reovirus causes the necrosis of hepatopancreocytes and inflammatory cells in hepatopancreatic tubules [48]. Notably, we have recorded a similar condition, the acute necrotizing hepatopancreatitis, along the whole invasion range of the signal crayfish in the Korana River, which is currently classified as idiopathic [49]. Thus, future studies should examine the recorded signal crayfish-associated reo-like virus 1 as a potential causative agent of this disease.

**Figure 5:**
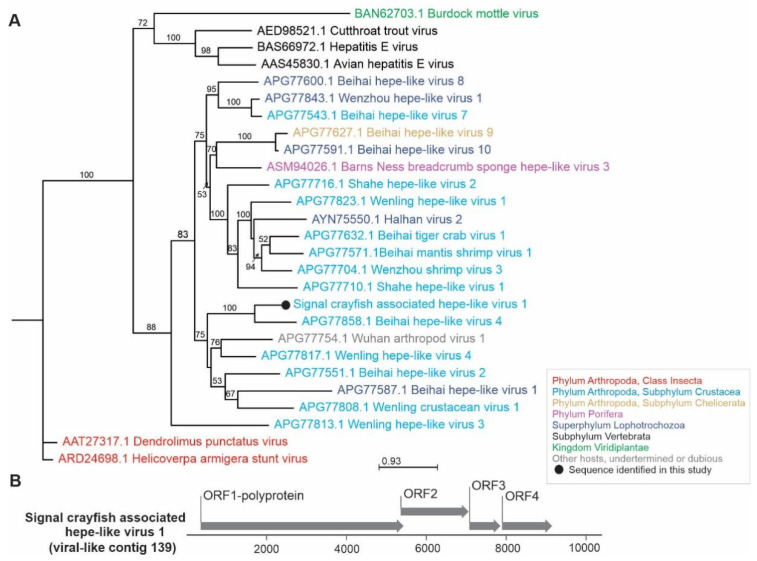
Phylogenetic relationships and genome organization of signal crayfish-associated hepe-like virus 1. (**A**) Phylogenetic tree built with maximum likelihood approach, based on the alignment of the conserved segment of RNA-dependent RNA polymerase domain of signal crayfish-associated hepe-like virus 1 (virus-like contig 139) and representative selected sequences of phylogenetically related viruses. The numbers on the branches represent bootstrap support values (>50% shown), the branch length represents the average number of amino acid substitutions per site. (**B**) The predicted genome organization of the novel signal crayfish-associated hepe-like virus 1, with positions and lengths of the open reading frames (ORF) indicated with corresponding arrow lengths.

Signal crayfish-associated hepe-like virus 1 (virus-like contig 139, 10,400 nts), detected in all samples, is represented by near complete genome sequence with four open reading frames (ORF), including ORF1—nonstructural polyprotein with RdRp domain (Figure 5). In blastx analysis, this contig had the highest (44%) amino acid identity to sequence of Beihai hepe-like virus 4, identified from the blue swimmer crab (deposited sequence is 11,635 nts long) and octopus (deposited sequence is 12,648 nts long) in China [3]. Consistent with the blastx search, phylogenetic analysis showed clustering of the detected virus sequence with Beihai hepe-like virus 4 and other hepe-like viruses from different crustacean hosts (Figure 5). Aside from reports in large-scale metagenomics studies [3], novel hepe-like virus has recently been characterized from the cephalothoraxes of the economically important giant freshwater prawn *Macrobrachium rosenbergii* with growth retardation, however, it could not be determined as the causative agent of the disease [50].

**Figure 6:**
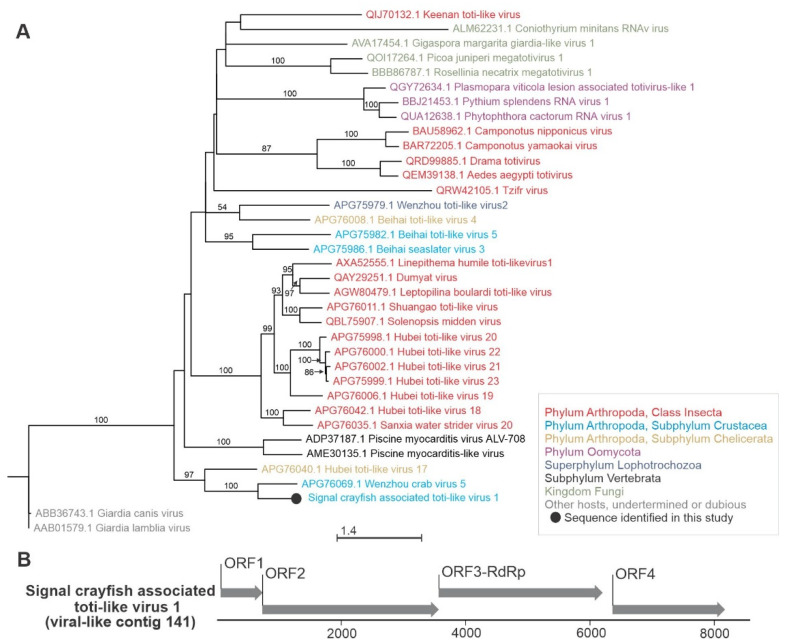
Phylogenetic relationships and genome organization of signal crayfish-associated toti-like virus 1. (**A**) Phylogenetic tree built with maximum likelihood approach, based on the alignment of the conserved segment of RNA-dependent RNA polymerase domain of signal crayfish-associated toti-like virus 1 (virus-like contig 141) and representative selected sequences of phylogenetically related viruses. The numbers on the branches represent bootstrap support values (>50% shown), the branch length represents the average number of amino acid substitutions per site. (**B**) The predicted genome organization of the novel signal crayfish-associated toti-like virus 1 with positions and lengths of the open reading frames (ORF) indicated with corresponding arrow lengths.

The signal crayfish-associated toti-like virus 1 (virus-like contig 141, 8576 nts), containing four ORFs, was clustered with the Wenzhou crab virus 5 (deposited sequence is 8691 nts long) in a phylogenetic analysis (Figure 6). It had a 41% amino acid identity in blastx analysis with the same virus, which was associated with the *Charybdis* crab host [3] and was detected in all of our samples. Similarities to other viruses, especially toti-like viruses, from different insect hosts were also detected using blastx. Other viruses from toti-like virus clade, with no blastx similarity to sequence identified in this study, and not included in phylogenetic analysis, are known to be associated with crayfish; infectious myonecrosis virus (IMNV), the causative agent of infectious myonecrosis in the Pacific white shrimp (*Litopenaeus vannamei*) [51], and the Cherax giardiavirus-like virus (CGV) in freshwater crayfish (*Cherax quadricarinatus*) [52]. However, IMNV targets mesoderm-derived tissues, like muscles, connective tissue, and hemocytes, and does not replicate in enteric tissues like hepatopancreas [53]. CGV was, however, reported by histology in hepatopancreas, similar to the toti-like virus identified in this study, but it has been neither sequenced nor isolated [52,53].

**Figure 7:**
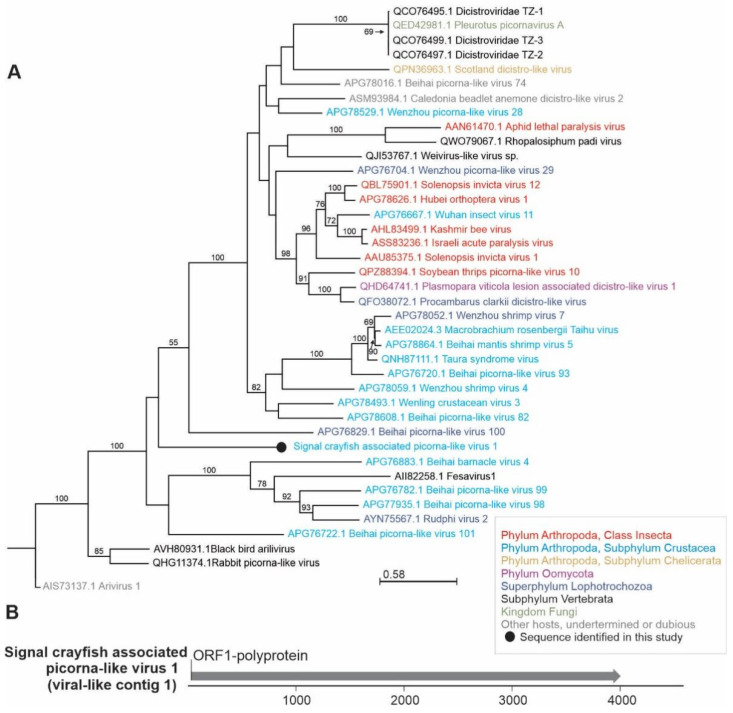
Phylogenetic relationships and genome organization of signal crayfish-associated picorna-like virus 1. (**A**) Phylogenetic tree built with maximum likelihood approach, based on the alignment of the conserved segment of RNA-dependent RNA polymerase domain of signal crayfish-associated picorna-like virus 1 (virus-like contig 1) and representative selected sequences of phylogenetically related viruses. The numbers on the branches represent bootstrap support values (>50% shown), the branch length represents the average number of amino acid substitutions per site. (**B**) The predicted partial genome organization of the novel signal crayfish-associated picorna-like virus 1, with positions and length of the open reading frame (ORF) indicated with corresponding arrow length.

Distinct contigs similar to previously described picorna-like viruses, a loosely defined broad group of viruses, were detected mostly in UF, with the highest blastx similarities to different crustacean picorna-like viruses (Table 2), such as Changjiang picorna-like virus 6, Beihai picorna-like virus 99, and Wenzhou picorna-like virus 38 [3]. The longest (4587 nts) picorna-like virus contig (virus-like contig 1, signal crayfish-associated picorna-like virus 1) with the highest read coverage had 26% amino acid identity in the blastx analysis with the Beihai picorna-like virus 99 (deposited sequence is 6503 nts long) isolated from a hermit crab in China [3]. Based on phylogenetic analysis and genome organization (Figure 7), with ORF1 nonstructural polyprotein comprising RdRp domain, signal crayfish-associated picorna-like virus 1 represents a single-sequence branch placed in the cluster that contains viruses of different invertebrate hosts, including crustacean viruses. In the same cluster, Taura syndrome virus is a known disease-causing crustacean virus that has spread rapidly throughout shrimp farming regions globally [23], and was reported to induce significant changes in the hepatopancreas transcriptome of the Pacific white shrimp [54]. Besides most similar viruses included in our phylogenetic analysis, mud crab dicistrovirus has been described previously as pathogenic to the mud crab (*Scylla paramamosain*) [55]. Other sequences distantly similar to picorna-like viruses (virus-like contigs 10, 11, 15, 9, 5, 13, 116, 2, 36) were also identified with low average read coverage, and could not be further assembled into longer contigs.

Further viral contigs potentially associated with signal crayfish, often detected in only one sample and with low average read coverage, belonged to tombus-like, chu-like, partiti-like, sobemo-like, and narna-like virus groups. Sequences similar to tombus-like viruses were previously associated with plant hosts, however, numerous tombus-related viruses have recently been described in non-plant hosts, such as marine and terrestrial invertebrates. Based on the phylogenetic analyses, it was suggested that this group of viruses was primarily associated with invertebrates, and was later horizontally transferred from aquatic invertebrates to plant hosts [3,6]. Apart from HTS based studies, tombus-like viruses have not been widely studied as infectious agents in crustacean hosts, except for the mud crab tombus-like virus that was associated with the sleeping disease in crabs, but only as a part of co-infection with mud crab dicistrovirus [56]. In this study, distinct tombus-like virus sequences were detected in signal crayfish hepatopancreas. Signal crayfish-associated tombus-like virus 1 (virus-like contig 66, 4504 nts) with a near complete genome sequence found in the DC location was most similar to the Caledonia beadlet anemone tombus-like virus 1 (41% identity on amino acid level) isolated from the anemone species *Actinia equine* [57]. Signal crayfish-associated tombus-like virus 2 (virus-like contig 84, 2981 nts) was detected in the DF location and was also most similar to Caledonia beadlet anemone tombus-like virus 1 (39% identity on amino acid level) [57]. These two tombus-like virus contigs had an average read coverage higher than ten, in contrast to lower read coverage of additional tombus-like virus contigs (virus-like contigs 55, 35, 169) (1425, 665, 301 nts respectively) detected in the UC location with the highest blastx-based identities with different invertebrate and plant infecting tombus-like viruses. Low read coverage tombus-like contigs may represent plant-infecting viruses, derived from food sources, while those with higher read coverage are more likely to be true crayfish viruses.

At all locations, different contigs similar to the Beihai hermit crab virus 3 [3] were identified. Virus-like contig 140 (signal crayfish-associated chu-like virus 1, 2216 nts) was the longest, and had a 27% amino acid sequence identity with hypothetical protein 2. Virus-like contig 145 (signal crayfish-associated chu-like virus 4, 1009 bp) had a 30% amino acid sequence identity to RdRp. The Beihai hermit crab virus 3 belongs to the *Mivirus* genus from the recently discovered negative-strand RNA *Chuviridae* family, characterized by HTS analysis only [3,31,58]. Diverse chuvirus-derived endogenous viral elements were also detected in mosquito genomes [59]. Additional contigs (virus-like contigs 65, 7, 146, 222) exhibited similarity with the Beihai hermit crab virus 3 hypothetical protein 2 or RdRp, but could not be further assembled into longer contigs, and did not contain full-length RdRp domain. In the UF location we have also detected a signal crayfish-associated partiti-like virus 1 (virus-like contig 27, 1188 nts) with a 56% amino acid identity to Caledonia partiti-like virus isolated from the breadcrumb sponge (*Halichondria panacea*) [57]. According to the blastx results, signal crayfish-associated partiti-like virus 1 was also similar to other crustacean-associated viruses (Wenling partiti-like virus 13, Wenling partiti-like virus 11) [3] and may with relatively high average read coverage (28) represent a crayfish-infecting virus.

### 3.2. Abundance of Viral Sequences and Nucleotide Diversity of Selected Virus Populations along the Invasion Range

We have compared the abundance of viral sequences as well as the nucleotide diversity of selected virus populations at different sampling locations in order to obtain insights into the possible patterns of variations along the invasion range. Based on the observed patterns, we could postulate hypotheses about how the observed patterns could be connected with ecological conditions at different sample sites, which represent a baseline for future research work on the virus ecology of crayfish and/or invasive alien species.

In our study, an abundance of virus-like reads, distinct virus-like contigs, and sequences mapping to signal crayfish-associated virus-like contigs varied between locations along the invasion range, with virus-like reads constituting between 0.0016% of the total reads in UF and 0.0174% in DC (Table 1). When comparing the two upstream and two downstream samples, the ones at the invasion cores had higher numbers of virus-like reads detected in comparison to those from the corresponding front sites. In addition, higher read coverage of viral sequences belonging to two out of three of the most represented newly identified signal crayfish-associated viruses was observed in the invasion cores compared to invasion fronts (Figure 2). At the same time, higher crayfish abundances were observed at the invasion cores (Figure 1).

The differences in signal crayfish abundance along the invasion range could influence the abundance of virus sequences detected, according to the classical epidemiological theory that links larger populations with higher contact rates with an increased likelihood of viral transmission [60,61,62]. The UF location differed the most from other locations in several aspects. The lowest percentage of virus-like reads was detected, and also lower reads coverages for signal crayfish-associated reo-like virus 1 (virus-like contig 4), crayfish-associated hepe-like virus 1 (virus-like contig 139), and toti-like virus 1 (virus-like contig 141) were found for this locality (Figure 2). Also, the lowest crayfish abundance was estimated at this locality, which probably resulted in lower encounter rates between individuals. Additionally, in the UF, we have recorded a lower severity of the acute necrotizing hepatopancreatitis [49]. This correlates with above mentioned lower read abundance at this location, and implies that some of the detected putative novel viruses in this study may be associated with the observed pathological changes in hepatopancreas. To test this hypothesis, further studies would be needed to associate individual crayfish samples of a known health status with the presence of putative novel viruses discovered in this study.

Despite the lower total number of virus-like reads in the UF sample, the highest number of distinct putative virus-like contigs similar to unclassified RNA viruses (Table S2) was found here, and picorna-like virus contigs were found predominantly in this sample. According to previous research [63,64,65], a decrease in viral diversification is expected in less dense groups, such as invasion fronts. In contrast, we observed the highest number of distinct viral contigs in the UF location, a site with the lowest crayfish abundance. However, at invasion fronts, the population of the native *P. leptodactylus* is also present with 2.5–12.9 higher abundances than the signal crayfish [27], potentially contributing to the inter-specific transmission of viruses. The observed differences in the number of total virus-like reads between the samples could also partially result from methodological limitations, such as difficulties in detecting highly divergent viral sequences that are considerably different from the reference genomes of known viruses [66].

**Figure 8:**
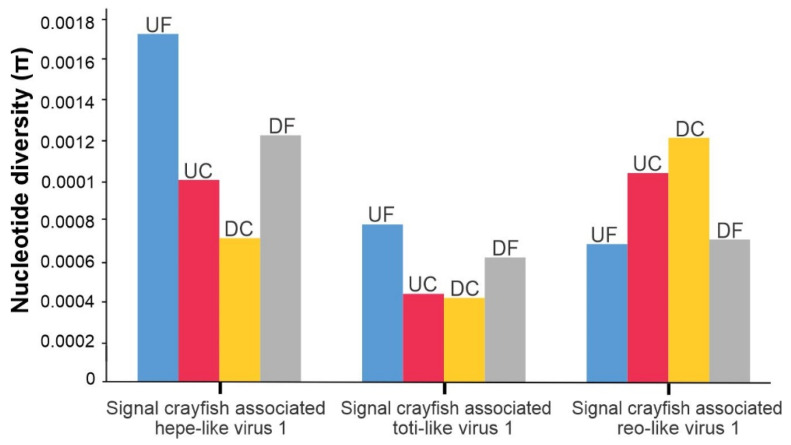
Nucleotide diversity values (π) calculated for signal crayfish-associated hepe-like virus 1, signal crayfish-associated toti-like virus 1, and signal crayfish-associated reo-like virus 1 at different locations (UF—upstream front, UC—upstream core, DC—downstream core, DF—downstream front) along the invasion range of the signal crayfish in the Korana River, Croatia.

For the three viruses, which were present at all four locations with relatively high average read coverage, we determined the diversities of their populations by calculating nucleotide diversities. This enabled us to estimate how the diversity of each discovered viral species population might be changing along its host invasion range. For all analyzed viruses, we observed differences in nucleotide diversity between populations at the invasion cores and invasion fronts. We observed two contrasting patterns. For signal crayfish-associated reo-like virus 1, the nucleotide diversity was decreased at the invasion fronts, while for signal crayfish-associated hepe-like virus 1 and toti-like virus 1, the nucleotide diversity was higher at the invasion fronts (Figure 8).

Observed differences in nucleotide diversities of viral populations of the three investigated viruses from samples collected along the signal crayfish invasion range might result from different processes, and several hypotheses can be put forward to explain the observed patterns. For instance, higher nucleotide diversity might indicate a higher prevalence of a particular virus, e.g., reo-like virus in the core sites. However, since we were analyzing composite hepatopancreas samples, we are limited in our estimations of the prevalence of individual viruses. Furthermore, we speculate that the estimated nucleotide diversity (π) values potentially reflect transmission dynamics of analyzed viruses, with a higher core nucleotide diversity of signal crayfish-associated reo-like virus 1 suggesting the transmission of the virus from longer established core populations with high signal crayfish abundance to recently established front populations of low signal crayfish abundance. The signal crayfish-associated reo-like virus 1 might be an endemic virus of signal crayfish, introduced to the new habitat along with its host, which could be confirmed/rejected by detecting the signal crayfish-associated reo-like virus 1 sequence in signal crayfish from other geographic regions. On the contrary, a higher nucleotide diversity of signal crayfish-associated toti-like virus 1 and hepe-like virus 1 in samples from invasion fronts could potentially reflect the introduction of these viruses from other host populations (e.g., from the native *P. leptodactylus* with higher density at the fronts) to populations of low signal crayfish abundance at invasion fronts. Successful inter-specific transmission relies on physical opportunities for transmission, such as occupying the same environment at the same time [67]. At invasion cores, the signal crayfish have displaced the native host, which would, under the proposed scenario, explain the decrease in nucleotide diversity for signal crayfish-associated toti-like virus 1 and hepe-like virus 1. However, such host associations should be further investigated, since very few of the crustacean viruses have been tested for infectivity in species other than the original host [68]; this hypothesis could be tested by analyzing native crayfish species for the presence of signal crayfish-associated toti-like virus 1 and hepe-like virus 1. Another possible hypothesis for the observed patterns might be a non-random dispersal of the host individuals during the invasion process, as demonstrated for a number of invasive species (i.e., [69]).

Finally, the results regarding the comparison of different locations can be confounded by other factors, which we were not able to elucidate using our experimental design (sequencing of samples pooled by location), and further analysis of individual samples would be needed to advance the understanding and test the postulated hypotheses.

## 4. Conclusions

- We reported, for the first time, the virome of signal crayfish hepatopancreas tissue and found a high diversity of novel divergent viral sequences most similar to different unclassified RNA viruses.
- We identified putative novel RNA viruses, including near complete genome sequence of signal crayfish-associated hepe-like virus 1 and toti-like virus 1, and the partial genomes of signal crayfish-associated reo-like virus 1 and picorna-like viruses. We identified additional tombus-like, partiti-like, and chu-like virus sequences potentially representing novel crayfish viruses. This pioneer study represents a baseline for the future research of a signal crayfish virome, e.g., to confirm the association of novel viruses with signal crayfish host, and to investigate their potential involvement in the observed necrotizing hepatopancreatitis.
- We speculate that the differences in the signal crayfish population density along the invasion range, non-random dispersal, and possibilities of inter-specific viral transmissions may have an effect on the diversity and abundance of signal crayfish-associated viral sequences. Different hypotheses can be postulated to explain these patterns, and this study represents a baseline for the further research of virus transmission dynamics as a result of the invader’s fast dispersal, including inter-species transmission between the signal crayfish as an invader and *P. leptodactylus* as a co-occurring and phylogenetically related native species.

## Figures and Tables

**Table 1:** Taxonomic classification of reads obtained from different locations (UF—upstream front, UC—upstream core, DC—downstream core, DF—downstream front).

Sample Name	Raw Reads	Reads after Trimming	Accession Number (SRA *)	% of Classified Reads (Diamond)	% of Reads Class. as Viral (Diamond)	% of Reads Class. as *Riboviria* (Diamond)	No. of Viral Read Class. as *Riboviria* (Diamond)
UF	164,043,698	160,643,914	SAMN20800941	15.47	0.00159	0.00094	1511
UC	149,357,758	146,166,391	SAMN20800942	19.06	0.00895	0.00816	11,929
DC	100,131,420	97,563,944	SAMN20800943	23.24	0.01737	0.01620	15,802
DF	136,134,398	132,904,626	SAMN20800944	15.29	0.00961	0.00821	10,905

* short reads archive (SRA).

**Table 2:** Selected putative invertebrate virus-like contigs (>300 nts) representing signal crayfish-associated viruses identified in this study with their contig length (nts) and closest match in the blastx (NCBI-nr) analysis.

Contig Name	Virus Name	GenBank Accesion	Contig Length	Blastx Results		
				Closest Protein Hit Name and Species	e-Value	Identities *
**Virus-like contig 4**	Signal crayfish associated reo-like virus 1	OK317706	4234	RdRp [Cherax quadricarinatus reovirus]	2.74 × 10^−20^	106/422 (25%)
**Virus-like contig 139**	Signal crayfish associated hepe-like virus 1	OK317707	10,400	hypothetical protein [Beihai hepe-like virus 4]	2.64 × 10^−117^	213/480 (44%)
**Virus-like contig 141**	Signal crayfish associated toti-like virus 1	OK317708	8576	hypothetical protein 4 [Wenzhou crab virus 5]	<1.00 × 10^−250^	353/869 (41%)
**Virus-like contig 1**	Signal crayfish associated picorna-like virus 1	OK317711	4587	hypothetical protein [Beihai picorna-like virus 99]	1.28 × 10^−86^	324/1251 (26%)
**Virus-like contig 10**	Signal crayfish associated picorna-like virus 2	OK317712	531	hypothetical protein [Beihai picorna-like virus 99]	2.75 × 10^−39^	79/175 (45%)
**Virus-like contig 11**	Signal crayfish associated picorna-like virus 3	OK317713	320	hypothetical protein [Beihai picorna-like virus 99]	4.97 × 10^−51^	80/105 (76%)
**Virus-like contig 15**	Signal crayfish associated picorna-like virus 4	OK317714	898	hypothetical protein 1 [Changjiang picorna-like virus 6]	1.67 × 10^−37^	85/151 (56%)
**Virus-like contig 9**	Signal crayfish associated picorna-like virus 5	OK317715	771	hypothetical protein [Beihai picorna-like virus 99] Y	1.30 × 10^−96^	170/257 (66%)
**Virus-like contig 5**	Signal crayfish associated picorna-like virus 6	OK317716	714	hypothetical protein [Wenzhou picorna-like virus 38]	1.56 × 10^−19^	62/152 (41%)
**Virus-like contig 13**	Signal crayfish associated picorna-like virus 7	OK317717	1894	hypothetical protein [Beihai sesarmid crab virus 2]	9.08 × 10^−99^	226/642 (35%)
**Virus-like contig 66**	Signal crayfish associated tombus-like virus 1	OK317718	4504	replicase [Caledonia beadlet anemone tombus-like virus 1]	<1.00 × 10^−250^	352/862 (41%)
**Virus-like contig 84**	Signal crayfish associated tombus-like virus 2	OK317719	2981	replicase [Caledonia beadlet anemone tombus-like virus 1]	3.00 × 10^−114^	246/628 (39%)
**Virus-like contig 55**	Signal crayfish associated tombus-like virus 3	OK317720	1425	RdRp [Riboviria sp.]	2.00 × 10^−92^	150/299 (50%)
**Virus-like contig 35**	Signal crayfish associated tombus-like virus 4	OK317721	665	RdRp [Riboviria sp.]	4.00 × 10^−32^	76/161 (47%)
**Virus-like contig 24**	Signal crayfish associated tombus-like virus 5	OK317722	538	hypothetical protein 1 [Hubei tombus-like virus 16]	3.98 × 10^−04^	43/99 (43%)
**Virus-like contig 169**	Signal crayfish associated tombus-like virus 6	OK317723	301	hypothetical protein 2 [Hubei unio douglasiae virus 2]	2.00 × 10^−42^	69/82 (84%)
**Virus-like contig 140**	Signal crayfish associated chu-like virus 1	OK317724	2216	hypothetical protein 2 [Beihai hermit crab virus 3]	5.71 × 10^−56^	161/588 (27%)
**Virus-like contig 65**	Signal crayfish associated chu-like virus 2	OK317725	746	hypothetical protein 2 [Beihai hermit crab virus 3]	6.45 × 10^−35^	69/167 (41%)
**Virus-like contig 7**	Signal crayfish associated chu-like virus 3	OK317726	493	RdRp [Beihai hermit crab virus 3]	3.98 × 10^−48^	44/54 (81%)
**Virus-like contig 145**	Signal crayfish associated chu-like virus 4	OK317727	1009	RdRp [Beihai hermit crab virus 3]	1.63E × 10^−60^	120/219 (55%)
**Virus-like contig 146**	Signal crayfish associated chu-like virus 5	OK317728	418	RdRp [Beihai hermit crab virus 3]	1.00E × 10^−27^	54/98 (55%)
**Virus-like contig 222**	Signal crayfish associated chu-like virus 6	OK317729	356	RdRp [Beihai hermit crab virus 3]	2.00E × 10^−45^	80/111 (72%)
**Virus-like contig 27**	Signal crayfish associated partiti-like virus 1	OK317730	1188	RdRp [Caledonia partiti-like virus]	1.00E × 10^−142^	221/394 (56%)
**Virus-like contig 116**	Signal crayfish associated picorna-like virus 8	OK317731	734	polyprotein [Picornaviridae sp.]	3.70E × 10^−10^	42/88 (48%)
**Virus-like contig 2**	Signal crayfish associated picorna-like virus 9	OK317732	412	hypothetical protein 1 [Picornavirales sp.]	1.05E × 10^−24^	45/100 (45%)
**Virus-like contig 36**	Signal crayfish associated picorna-like virus 10	OK317733	457	RdRp [Picornavirales sp.]	4.32 × 10^−16^	60/102 (58%)
**Virus-like contig 83**	Signal crayfish associated sobemo-like virus 1	OK317734	431	hypothetical protein [Hubei sobemo-like virus 43]	1.00 × 10^−50^	84/143 (59%)
**Virus-like contig 147**	Signal crayfish associated sobemo-like virus 2	OK317709	512	hypothetical protein 1 [Beihai sobemo-like virus 17]	1.00E × 10^−10^	44/128 (34%)
**Virus-like contig 30**	Signal crayfish associated narna-like virus 1	OK317710	378	RdRp [Beihai narna-like virus 18]	5.60 × 10^−18^	48/128 (38%)

* number of matching amino acids/number of total amino acids of the closest protein hit (percentage of amino acid identity).

## Data Availability

All sequencing data that support the findings of this study are linked under the accession number PRJNA754774 in the NCBI BioProject database (https://www.ncbi.nlm.nih.gov/sra, accessed on 9 November 2021). The sequences of selected viral contigs identified in this study are available in Table S2 and have been deposited in the GenBank under the accession numbers OK317706-OK317734.

## Supplementary Materials

The following are available online at https://www.mdpi.com/article/10.3390/v13112259/s1, Figure S1. Schematic representation of different steps of the bioinformatic analysis of sequencing reads (black arrows) and contigs (green arrows) to identify virus-like sequences in the signal crayfish hepatopancreas samples; Table S1. Selected putative invertebrate virus-like contigs (>300 nts) representing signal crayfish associated viruses identified in this study with their contig length (nts) and percentage of mapped reads resulting from mapping of reads from individual samples to virus-like contigs; Table S2. (A) Virus-like contigs from different locations (UF—upstream front, UC—upstream core, DC—downstream core, DF—downstream front) identified in Diamond analysis together with average coverage values, Diamond classification, blastn similarity search results (NCBI-nt, April 2021) and sequences of individual virus-like contigs. (B) virus-like contigs from different locations (UF—upstream front, UC—upstream core, DC—downstream core, DF—downstream front) identified using pfam domain search, where the remaining contigs not classified as viral by Diamond (963721) were translated and compared with the entire Pfam database, together with average coverage values, Pfam search results and blastn (NCBI-nt, April 2021) and blastx (NCBI-nr, June 2021) similarity search results; Table S3. Sequences of primers used for PCR amplification of selected virus-like contigs representing signal crayfish associated viruses identified in this stud; Alignment S1. Sequence alignments (.fasta) with virus names and corresponding GenBank accession numbers of viruses used for phylogenetic analysis of signal crayfish associated reo-like virus 1; Alignment S2. Sequence alignments (.fasta) with virus names and corresponding GenBank accession numbers of viruses used for phylogenetic analysis of signal crayfish associated hepe-like virus 1; Alignment S3. Sequence alignments (.fasta) with virus names and corresponding GenBank accession numbers of viruses used for phylogenetic analysis of signal crayfish associated toti-like virus 1; Alignment S4. Sequence alignments (.fasta) with virus names and corresponding GenBank accession numbers of viruses used for phylogenetic analysis of signal crayfish associated picorna-like virus 1.
