# Supplementary material for: Virome Analysis of Signal Crayfish (Pacifastacus leniusculus) along Its Invasion Range Reveals Diverse and Divergent RNA Viruses
Source: Viruses. 2021 Nov 11;13(11):2259. doi: 10.3390/v13112259 (PMC8624288; doi:10.3390/v13112259)
Supplement: Supplementary file 1 [file viruses-13-02259-s001.zip › Supplementary_Bacnik_revisions.pdf]

viruses

MDPI

Supplementary file

Virome analysis of signal crayfish *(Pacifastacus leniusculus)* along its invasion range reveals diverse and divergent RNA viruses

Katarina Bačnik, Denis Kutnjak, Silvija Černi, Ana Bielen and Sandra Hudina

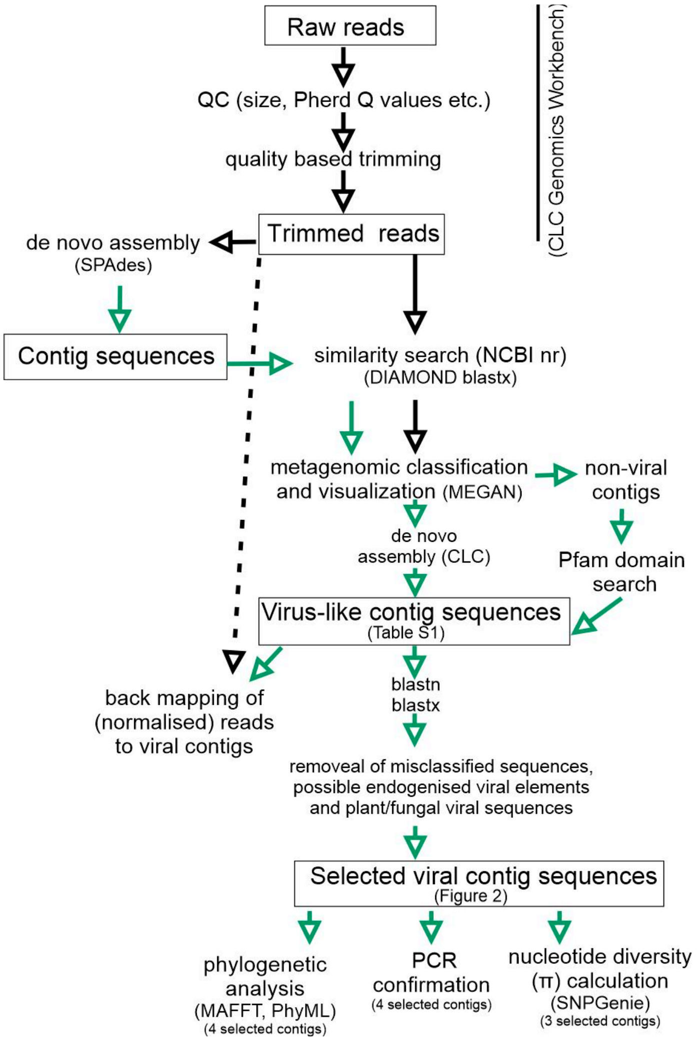

Figure S1. Schematic representation of different steps of the bioinformatic analysis of sequencing reads (black arrows) and contigs (green arrows) to identify virus-like sequences in the signal crayfish hepatopancreas samples

Table S1. Selected putative invertebrate virus-like contigs (> 300 nts) representing signal crayfish associated viruses identified in this study with their contig length (nts) and percentage of mapped reads resulting from mapping of reads from individual samples to virus-like contigs.

				**Percentage of mapped reads (%)**			
**Contig name**	**Virus name**	**GenBank accesion**	**Contig length**	**UF**	**UC**	**DC**	**DF**
**Virus-like contig 4**	Signal crayfish associated reo-like virus 1	OK317706	4,234	0.035179	0.793427	0.334182	0.451408
**Virus-like contig 139**	Signal crayfish associated hepe-like virus 1	OK317707	10,400	0.003373	0.322649	0.609349	0.300643
**Virus-like contig 141**	Signal crayfish associated toti-like virus 1	OK317708	8,576	0.010525	0.023344	0.025111	0.019544
**Virus-like contig 1**	Signal crayfish associated picorna-like virus 1	OK317711	4,587	0.000300	0.000003	0.000000	0.000000
**Virus-like contig 10**	Signal crayfish associated picorna-like virus 2	OK317712	531	0.000014	0.000000	0.000000	0.000000
**Virus-like contig 11**	Signal crayfish associated picorna-like virus 3	OK317713	320	0.000004	0.000000	0.000000	0.000000
**Virus-like contig 15**	Signal crayfish associated picorna-like virus 4	OK317714	898	0.000022	0.000005	0.000000	0.000001
**Virus-like contig 9**	Signal crayfish associated picorna-like virus 5	OK317715	771	0.000016	0.000000	0.000000	0.000000
**Virus-like contig 5**	Signal crayfish associated picorna-like virus 6	OK317716	714	0.000032	0.000000	0.000000	0.000000
**Virus-like contig 13**	Signal crayfish associated picorna-like virus 7	OK317717	1,894	0.000065	0.000000	0.000000	0.000000
**Virus-like contig 66**	Signal crayfish associated tombus-like virus 1	OK317718	4,504	0.000000	0.000001	0.000380	0.000000
**Virus-like contig 84**	Signal crayfish associated tombus-like virus 2	OK317719	2,981	0.000000	0.000000	0.000000	0.000220
**Virus-like contig 55**	Signal crayfish associated tombus-like virus 3	OK317720	1,425	0.000000	0.000047	0.000000	0.000000
**Virus-like contig 35**	Signal crayfish associated tombus-like virus 4	OK317721	665	0.000000	0.000021	0.000000	0.000000
**Virus-like contig 24**	Signal crayfish associated tombus-like virus 5	OK317722	538	0.000009	0.000000	0.000000	0.000000
**Virus-like contig 169**	Signal crayfish associated tombus-like virus 6	OK317723	301	0.000000	0.000002	0.000000	0.000000
**Virus-like contig 140**	Signal crayfish associated chu-like virus 1	OK317724	2,216	0.000049	0.000170	0.000099	0.000099
**Virus-like contig 65**	Signal crayfish associated chu-like virus 2	OK317725	746	0.000002	0.000003	0.000017	0.000003
**Virus-like contig 7**	Signal crayfish associated chu-like virus 3	OK317726	493	0.000006	0.000003	0.000002	0.000003
**Virus-like contig 145**	Signal crayfish associated chu-like virus 4	OK317727	1,009	0.000111	0.000094	0.000071	0.000147
**Virus-like contig 146**	Signal crayfish associated chu-like virus 5	OK317728	418	0.000002	0.000003	0.000003	0.000006
**Virus-like contig 222**	Signal crayfish associated chu-like virus 6	OK317729	356	0.000001	0.000000	0.000000	0.000005
**Virus-like contig 27**	Signal crayfish associated partiti-like virus 1	OK317730	1,188	0.000247	0.000000	0.000000	0.000000
**Virus-like contig 116**	Signal crayfish associated picorna-like virus 8	OK317731	734	0.000000	0.000000	0.000000	0.000012
**Virus-like contig 2**	Signal crayfish associated picorna-like virus 9	OK317732	412	0.000007	0.000000	0.000000	0.000000
**Virus-like contig 36**	Signal crayfish associated picorna-like virus 10	OK317733	457	0.000001	0.000005	0.000000	0.000002
**Virus-like contig 83**	Signal crayfish associated sobemo-like virus 1	OK317734	431	0.000000	0.000000	0.000000	0.000012
**Virus-like contig 147**	Signal crayfish associated sobemo-like virus 2	OK317709	512	0.000009	0.000000	0.000000	0.000004
**Virus-like contig 30**	Signal crayfish associated narna-like virus 1	OK317710	378	0.000004	0.000000	0.000000	0.000000

Table S2. (A) Virus-like contigs from different locations (UF – upstream front, UC – upstream core, DC – downstream core, DF – downstream front) identified in Diamond analysis together with average coverage values, Diamond classification, blastn similarity search results (NCBI-nt, April 2021) and sequences of individual virus-like contigs. (B) virus-like contigs from different locations (UF – upstream front, UC – upstream core, DC – downstream core, DF – downstream front) identified using pfam domain search, where the remaining contigs not classified as viral by Diamond (963721) were translated and compared with the entire Pfam database, together with average coverage values, Pfam search results and blastn (NCBI-nt, April 2021) and blastx (NCBI-nr, June 2021) similarity search results.

Table S3. Sequences of primers used for PCR amplification of selected virus-like contigs representing signal crayfish associated viruses identified in this study.

Virus-like Contig	Primer sequence (5'->3')	Orientation	Amplicon length
Virus-like contig 4 (signal crayfish associated reo-like virus 1)	TTCTGGCGCGACTTTAGCTT	forward	269
	GCCGCACGTTGCTGTAAATA	reverse	
Virus-like contig 139 (signal crayfish associated hepe-like virus 1)	GGTGACGACCTTGCGATCAT	forward	248
	GCGCCGCATAAGTGAATAGC	reverse	
Virus-like contig 141 (signal crayfish associated toti-like virus 1)	GGTTGTTGCACATGAAGCGT	forward	249
	GCCGTAATGCAGCGTGTTAG	reverse	
Virus-like contig 1 (signal crayfish associated picorna-like virus 1)	CCACGCACACGAAAAACCAT	forward	193
	CATGGTAGATGGTGGGCTCC	reverse	

Alignment S1. Sequence alignments (.fasta) with virus names and corresponding GenBank accession numbers of viruses used for phylogenetic analysis of signal crayfish associated reo-like virus 1

Alignment S2. Sequence alignments (.fasta) with virus names and corresponding GenBank accession numbers of viruses used for phylogenetic analysis of signal crayfish associated hepe-like virus 1.

Alignment S3. Sequence alignments (.fasta) with virus names and corresponding GenBank accession numbers of viruses used for phylogenetic analysis of signal crayfish associated toti-like virus 1.

Alignment S4. Sequence alignments (.fasta) with virus names and corresponding GenBank accession numbers of viruses used for phylogenetic analysis of signal crayfish associated picorna-like virus 1.
